# Characterising percentage energy from ultra-processed foods by participant demographics, diet quality and diet cost: findings from the Seattle Obesity Study (SOS) III

**DOI:** 10.1017/S0007114520004705

**Published:** 2021-09-14

**Authors:** Shilpi Gupta, Chelsea M. Rose, James Buszkiewicz, Linda K. Ko, Jin Mou, Andrea Cook, Anju Aggarwal, Adam Drewnowski

**Affiliations:** 1 Center for Public Health Nutrition, Department of Epidemiology, University of Washington, Seattle, WA 98105, USA; 2 Division of Public Health Sciences, Department of Cancer Prevention, Fred Hutchinson Cancer Research Center, Seattle, WA 98109, USA; 3 Department of Health Services, University of Washington, Seattle, WA 98105, USA; 4 MultiCare Institute for Research & Innovation, Tacoma, WA 98405, USA; 5 Biostatistics Unit, Kaiser Permanente Washington Health Research Institute, Seattle, WA 98101, USA; 6 Department of Biostatistics, University of Washington, Seattle, WA 98195, USA

**Keywords:** Ultra-processed foods, Diet cost, Energy density, Nutrient density, HEI-2015, NRF_9.3_, Residential property values

## Abstract

Higher consumption of ‘ultra-processed’ (UP) foods has been linked to adverse health outcomes. The present paper aims to characterise percentage energy from UP foods by participant socio-economic status (SES), diet quality, self-reported food expenditure and energy-adjusted diet cost. Participants in the population-based Seattle Obesity Study III (*n* 755) conducted in WA in 2016–2017 completed socio-demographic and food expenditure surveys and the FFQ. Education and residential property values were measures of SES. Retail prices of FFQ component foods (*n* 378) were used to estimate individual-level diet cost. Healthy Eating Index (HEI-2015) and Nutrient Rich Food Index 9.3 (NRF_9.3_) were measures of diet quality. UP foods were identified following NOVA classification. Multivariable linear regressions were used to test associations between UP foods energy, socio-demographics, two estimates of food spending and diet quality measures. Higher percentage energy from UP foods was associated with higher energy density, lower HEI-2015 and NRF_9.3_ scores. The bottom decile of diet cost ($216·4/month) was associated with 67·5 % energy from UP foods; the top decile ($369·9/month) was associated with only 48·7 % energy from UP foods. Percentage energy from UP foods was inversely linked to lower food expenditures and diet cost. In multivariate analysis, percentage energy from UP foods was predicted by lower food expenditures, diet cost and education, adjusting for covariates. Percentage energy from UP foods was linked to lower food spending and lower SES. Efforts to reduce UP foods consumption, an increasingly common policy measure, need to take affordability, food expenditures and diet costs into account.

High consumption of ‘ultra-processed’ (UP) foods as defined by the NOVA classification scheme^(1)^ has been associated with a wide range of adverse health outcomes. Studies have pointed to positive associations between percentage energy from UP foods and risk of excess weight gain^(2,3)^, obesity^(4)^, diabetes^(5)^, the metabolic syndrome^(6)^, hypertension^(7)^, depressive symptoms^(8,9)^, incident frailty^(10)^, cancer^(11)^ and all-cause mortality^(12,13)^. The global obesity pandemic^(14)^ was blamed on the rising consumption of industrial UP foods.

What many UP foods seem to have in common is low per energy cost^(15)^. In one study based on FFQ component foods^(15)^, UP foods cost $0·55 per 418 kJ compared with $1·45/418 kJ for minimally processed foods. A clinical study^(2)^ reported that the ingredients for 8368 kJ/d of UP meals cost $106/week, whereas unprocessed meals cost as much as $151/week or 42 % more, based on food prices at a local supermarket chain. The monetary cost of observed diets with high percentage of energy from UP foods still remains to be explored. None of the cited studies on the links between UP foods and health outcomes has addressed the relative affordability of UP foods relative to healthier and more ‘prudent’ options, a fundamental issue in all studies of social disparities, diets and health.

It is by now well established that food prices and diet costs contribute to the observed socio-economic disparities in diet quality and may affect health outcomes^(16–19)^. Low-cost energy-dense foods composed of refined grains, added sugars and added fats are generally more affordable than are the recommended diets of minimally processed lean meats and fish, fresh vegetables and fruit, or home-made pasta and bread^(16,17)^. The observed associations between lower socio-economic status (SES) and higher rates of obesity and other non-communicable diseases may be mediated, in part, by the low-cost and high reward value of processed energy-dense foods^(16–19)^.

The present analysis used data from the Seattle Obesity Study III (SOS III) to link percentage energy from UP foods to two measures of food spending: self-reported food expenditures and estimated individual-level diet costs. We expected that percentage energy from UP foods would be inversely linked to diet cost calculated per 8368 kJ^(15)^. A secondary aim was to compare percentage energy from UP foods by socio-demographic strata^(18)^. The present hypothesis was that percentage energy from UP foods would be higher among groups of lower education and incomes^(18)^ and living in more disadvantaged neighbourhoods. Processed energy-dense foods of minimal nutritional value often represent the lowest-cost option for the vulnerable low-income consumer^(18–20)^. Given that UP foods are primarily defined by their content of added fat, sugar and salt^(19)^, we expected an inverse correlation between percentage energy from UP foods and dietary nutrient density measures.

## Methods

### Study design and participants

The SOS III was a population-based longitudinal study of adult male and female residents of King, Pierce and Yakima Counties in WA State. Participant recruitment was county-specific, relying on address-based sampling schemes stratified by three bands of residential property values. For King County, property value ranges were <$199 000, $200 000–299 000 and $300 000+, following past SOS III protocols^(21)^. Potential participants were sent pre-notification letters and then contacted by phone to screen for eligibility. Participants were also recruited from lower-income neighbourhoods through community outreach to ensure broad representation by SES and race/ethnicity. Participant recruitment and data collection were conducted in-person by local staff at each research site (from July 2016 to May 2017).

Eligible adults were aged 21–59 years, household gatekeepers, not pregnant or breast-feeding (at the time of data collection), and without any mobility issues. Following initial verbal consent, participants were invited to complete the first in-person visit at local study site or at home (Yakima only). Written consent was provided during the in-person visit before starting the study procedures. Data were collected in English (in all three counties) and Spanish (in Yakima County). All study procedures were approved by the Institutional Review Boards of respective sites. The present analytical sample was based on 755 male and female respondents.

### Procedure and study variables

#### Computer-assisted health behaviour survey

A computer-based survey administered during an in-person interview was used to collect data on age, sex, race/ethnicity, household income, education, employment, marital status and household size. Data from county tax assessors at the tax parcel level for 2016 were used to estimate residential property value, as additional measure of SES^(21)^. Estimated monthly household food expenditures at home and away from home were obtained by self-report. At-home food expenditures included grocery purchases, whereas away-from-home food expenditures were on foods consumed outside home (restaurants and cafeteria). At-home and away-from-home expenditures were summed to create total monthly food expenditures variable. This was divided by household size to create total monthly food expenditures per capita.

#### Dietary intakes data

Dietary intakes data were collected using Fred Hutch FFQ also administered during the in-person interview. The FFQ consists of a list of 126 line-item foods that are visible to the respondent and 378 foods that are not. For purposes of nutrient analysis, each of the 126 line items is represented by a variable number of component foods that are weighted to calculate its energy and nutrient content.

The FFQ component items were aggregated into four NOVA food processing categories: unprocessed, processed, UP and culinary ingredients, using published classification schemes^(22)^. Unprocessed foods have been defined as those fresh, dry or frozen foods that had been subjected to minimal or no processing. The FFQ component foods included fresh meat, fish, fruits (such as apple, banana and apricots), salad, milk, vegetables (broccoli, green beans and potatoes), eggs, legumes and unsalted nuts (raisins and prunes) and seeds. Culinary ingredients were sugar, animal fats (butter) and oils (olive oil, rapeseed oil and maize oil), and salt^(22)^. Adding culinary ingredients (fat, sugar and salt) to wholesome fresh foods transformed them into processed foods. The FFQ component foods classified as processed foods included all kinds of cheese, ham, beer and wine. FFQ foods classified as UP foods included breads, jams and jelly, breakfast cereals, sweet snacks (cookies and cakes), pizza, potato chips or tortilla chips, soft drinks (sodas and fruit drinks), French fries, sauces (ketchup, mayonnaise), desserts (ice cream, frozen yogurt and sherbet) and, frozen meals, juices and soups.

### Energy-adjusted individual-level diet cost

Estimates of individual-level daily diet cost were obtained by joining dietary intake data from FFQ instruments with county-specific retail prices for 378 FFQ component foods. Retail prices were obtained from large supermarkets in King, Pierce and Yakima counties following standard and published procedures^(15,23)^.

Retail prices converted to dollars per 100 g edible portion were added to the G-SEL nutrient database. Effectively, food prices per 100 g were treated in the same way as energy density (kJ/100 g) or nutrient values, also expressed as amounts (g/mg per IU) per 100 g edible portion. Energy and nutrient content of the daily diet were obtained by summing all foods consumed by an individual on a given day. In an analogous manner, the estimated cost of the daily diet was obtained by summing the cost of all foods consumed. The G-SEL nutrient composition database was thus composed of forty-five energy and nutrient vectors and a single cost vector. The procedures of estimating diet costs from FFQ have been described previously^(16)^. The procedure has been used in studies conducted in France^(17)^, Spain^(24)^, UK^(25)^ and Japan^(26)^ and has become a part of the epidemiology toolbox. For analytical purpose, this diet cost was divided by energy intake and expressed per 8368 kJ/d (2000 kcal/d). Diet cost per d was then converted into monthly diet cost variable.

### Percentage energy from ultra-processed foods

NOVA classification guidelines^(1)^ separate foods into unprocessed, processed, UP and culinary ingredients. Unprocessed foods include fruits, vegetables, grains or meats that had been subjected to minimal or no processing. Culinary ingredients are defined as sugar, animal fats (butter) and vegetable oils, starches, salt, and vinegar. Processed foods are defined as having been manufactured by adding culinary ingredients to wholesome fresh foods. Examples include cheese, ham, salted, smoked, or canned meat, beer and wine. UP foods are defined as industrial creations that contain added fat, sugar and salt as well as ingredients not found in home cooking. Classified as UP foods are commercial breads (refined and whole grain), ready-to-eat breakfast cereals, cakes, sweet snacks, and pizza, French fries, soft drinks, ice cream, and frozen meals and soups.

The NOVA food classification^(1)^ was attached to each of 378 FFQ components foods in the G-SEL nutrient database as described above, to parallel nutrient values. In this way, a processing code was added to each of the 378 foods in the G-SEL database. The contribution of UP foods to energy and nutrients was then calculated for each SOS III participant. Dietary share of UP foods was computed by dividing the energy content intake from UP foods category with the total energy intake for individual diet.

### Dietary quality measures

Healthy Eating Index 2015 (HEI-2015) was developed to assess compliance with US 2015 dietary guidelines^(27,28)^. HEI-2015 score reflects an overall diet quality computed using nine adequacy components (total fruit, whole fruit, total vegetables, greens and beans, whole grains, dairy products, total protein, seafood and plant proteins and fatty acids) and four moderation components (refined grains, Na and saturated fat and added sugars). The HEI-2015 is a continuous score on the scale of 0–100 where higher score reflects higher diet quality.

The Nutrient Rich Food Index 9.3 (NRF_9.3_) was the second measure of dietary nutrient density^(29,30)^. The NRF_9.3_ score applied to total diets^(31)^ was based on nine nutrients to encourage (NR9 subscore) and three nutrients to limit (LIM subscore). Reference daily values were based on the US Food and Drug Administration and other standards^(30,31)^. The reference amounts were protein (50 g), fibre (28 g), vitamin A (900 mg), vitamin C (90 mg), vitamin D (20 μg), Ca (1300 mg), Fe (18 mg), K (4700 mg) and Mg (420 mg). The maximum recommended values for the LIM component were added sugar (50 g), saturated fat (20 g) and Na (2300 mg). The NRF_9.3_ was calculated as:
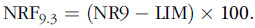



In NR9 calculation, each daily nutrient intake was adjusted for 8368 kJ and expressed in percentage of daily value. Following past protocol, percentage of daily values were truncated at 100 %, so that an excessively high intake of one nutrient could not compensate for the dietary inadequacy of another. In LIM, only the maximum recommended value share in excess of the recommended amount was considered.

### Statistical analysis

The present analysis made use of data from dietary intake assessment at baseline. Responses with missing data on socio-demographic variables, under and over-reporters of FFQ total energy intakes (<500 or >5000 kcal (<2092 or >20 920 kJ)) and extreme outliers on diet cost were excluded. The final analytic sample size was 755 individuals.

First, percentage of energy from UP foods was estimated for each participant. Analysis was conducted for the total sample and by socio-demographic group of interest. The mean and standard deviation for monthly diet cost (per 8368 kJ) was also calculated by each socio-demographic variable. Second, percentage of UP foods energy was also calculated by each cost indicator. A series of univariate linear regression models using generalised linear estimating equations with robust standard errors were used to test for significance across socio-demographic strata and cost indicators.

Third, percentage energy from UP foods and other dietary quality indicators were compared across tertiles of diet cost and monthly food expenditure. Individuals were classified by tertiles of monthly diet cost (adjusted per 8368 kJ). Mean and standard deviation for dietary share of UP foods, energy density, NRF subcomponents (NR9 and LIM) and HEI-2015 were compared across tertiles. Statistical tests of the association between cost tertiles, UP foods energy and diet quality measures were based on ANOVA.

Fourth, the association between UP foods energy and socio-demographic characteristics was tested using multiple-adjusted linear regression models with robust standard errors. UP foods energy was the dependent variable and sex, age, race, education, property value, self-reported food expenditure and diet cost were the independent variables. Model 1 was the multivariate model taking all the variables and covariates into account with the exception of self-reported expenditure. Model 2 was similar to model 1 but with the exception of diet cost. All statistical analyses were conducted using IBM SPSS Statistics for Windows, version 22.0 (IBM Corp. 2013).

## Results

Table 1 shows that the SOS III study sample was mostly female (82 %), married (58·5 %), evenly distributed by age group and with a high proportion of Hispanic participants. Whereas 44 % of the sample were college graduates, 34 % did not complete high school.

**Table 1.** Dietary share of ultra-processed foods and monthly diet costs (per 2000 kcal/d (8368 kJ)) by socio-demographic variables (Numbers and percentages; mean values and standard deviations)

Variables	Frequency		% Energy content from ultra-processed foods			Monthly diet cost/2000 kcal (8368 kJ)		
	*n*	%	Mean	sd	*P*	Mean	sd	*P*
Overall	755	100	59·68	10·73	–	283·66	59·41	–
Sex								
Male	135	17·9	58·43	10·46	Ref.	281·34	54·88	Ref.
Female	620	82·1	59·95	10·78	0·127	284·16	60·38	0·594
Age (years)								
21–40	286	37·9	60·24	11·13	Ref.	272·32	51·93	Ref.
41–50	230	30·5	60·57	9·95	0·721	287·07	62·08	0·004*
≥51	239	31·7	58·14	10·86	0·029*	293·94	63·03	<0·0001*
Education								
High school or less	255	33·8	64·12	8·46	Ref.	257·32	46·96	Ref.
Some college	166	22·0	60·22	10·28	<0·0001*	286·20	61·13	<0·0001*
College graduate/graduate school	334	44·2	56·01	11·18	<0·0001*	302·50	59·74	<0·0001*
Residential property value								
Tertile 1 (≤$128 675)	252	33·4	63·81	8·42	Ref.	257·48	46·02	Ref.
Tertile 2 ($128 676–$290 866)	253	33·5	58·97	11·10	<0·0001*	286·34	57·66	<0·0001*
Tertile 3 (≥$290 867)	250	33·1	56·22	11·09	<0·0001*	307·32	62·66	<0·0001*
Race/ethnicity								
Non-Hispanic White	366	48·5	57·67	10·96	Ref.	300·89	58·53	Ref.
Hispanic	307	40·7	63·27	8·82	<0·0001*	260·42	51·63	<0·0001*
Other	82	10·9	55·14	12·21	0·082	293·73	62·15	0·338
Marital status								
Married	442	58·5	59·38	10·81	0·371	286·54	59·57	0·108
Single	313	41·5	60·09	10·68	Ref.	279·54	59·04	Ref.

Ref., reference.

* Statistical significance at *P* < 0·05 and *P* < 0·0001.

Mean percentage of dietary energy from UP foods was 59·7 %. There were significant differences by socio-demographic strata. Higher percentage energy from UP foods was associated with younger adults (*P* < 0·029), Hispanic participants compared with non-Hispanic Whites (*P* < 0·0001), lower education (*P* < 0·0001) and lower residential property values (*P* < 0·0001). There were no significant effects of sex or marital status.

For each population subgroup, mean percentage energy from UP foods was inversely associated with FFQ-based estimates of energy-adjusted diet cost in $/month. Higher diet costs were associated with older adults, non-Hispanic Whites, college education and higher residential property values. There were no significant effects of sex or marital status.

Table 2 shows the relation between percentage energy from UP foods and two indicators of food spending: energy-adjusted diet costs and self-reported food expenditures, at home and total. Both variables were split into tertiles. An increase in diet cost was associated with a decline in UP foods energy from 65 to 53 %. An increase in food expenditures was associated with a decline in UP foods energy from 63 to 56 %.

**Table 2.** Dietary share of ultra-processed foods by food spending indicators (Numbers and percentages; mean values and standard deviations)

Variables	Frequency		% Energy content from ultra-processed foods		
	*n*	%	Mean	sd	*P*
Overall	755	100	59·68	10·73	–
Diet cost per 2000 kcal (8368 kJ) ($/month)					
≤$252·7	252	33·4	65·23	9·30	Ref.
≥$252·8 to ≤299·9	252	33·4	60·88	8·77	<0·0001*
≥$300	251	33·2	52·88	10·22	<0·0001*
Monthly food expenditures at home					
≤$100	276	36·6	62·84	9·97	Ref.
≥$101 to ≤175	237	31·4	59·45	10·11	<0·0001*
≥$176	242	32·1	56·28	11·12	<0·0001*
Total monthly food expenditures					
≤$144	252	33·4	62·63	10·08	Ref.
≥$145 to ≤250	271	35·9	59·85	10·07	0·002*
≥$251	232	30·7	56·26	11·21	<0·0001*

Ref., reference.

* Statistical significance at *P* < 0·01 and *P* < 0·0001.

**Fig. 1.**
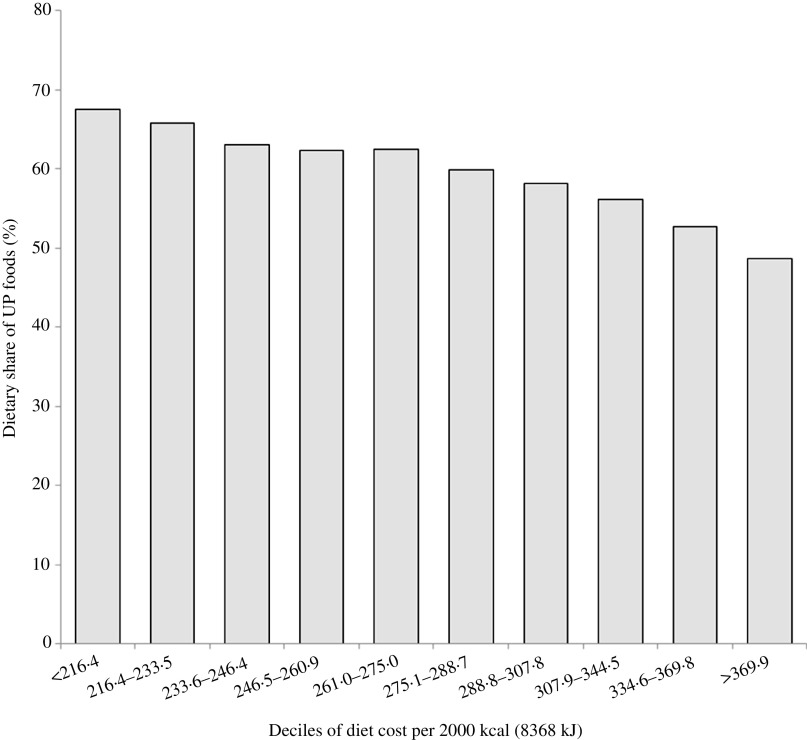
Dietary share of ultra-processed (UP) foods by deciles of diet cost ($) per 2000 kcal (8368 kJ).

Fig. 1 shows the inverse relation between percentage of energy from UP foods and deciles of energy-adjusted cost. The bottom decile of diet cost ($216·4/month) was associated with 67·5 % energy from UP foods; the top decile ($369·9/month) was associated with only 48·7 % energy from UP foods.

Table 3 shows multiple indicators of diet quality by tertiles of diet cost. As expected, the NRF_9.3_ dietary nutrient density score and its NR9 and LIM subcomponents showed a dose–response relation with diet cost. Dietary energy density was also a function of diet cost. Going from the bottom to the top tertile of diet cost, energy density of the diet, calculated based on foods and energetic beverages only, declined from 1·31 to 1·08 kcal/g (5·48 to 4·52 kJ/g). The LIM nutrient density subscore (based on saturated fat, added sugar and Na) decreased from 99·70 to 86·59 on going from the bottom to the top quartiles of diet cost. The NR9 based on protein, fibre, vitamins and minerals increased from 654·26 to 736·38. Total NRF_9.3_ scores increased as well. The mean HEI score increased by 9 points on going from the lowest quartile to the highest quartile of diet cost (62·57 *v*. 71·55). Table 3 also shows relation of diet quality indicators with tertiles of self-reported food expenditure. Similar to diet cost, mean HEI score also increased significantly points on going from the lowest quartile to the highest quartile of food expenditure (65·23 *v*. 69·45).

**Table 3.** Indicators of diet quality across tertiles (T) of estimated monthly diet cost (adjusted per 2000 kcal (8368 kJ)) and self-reported monthly food expenditure (Mean values and standard deviations)

	Tertiles of estimated monthly diet cost (adjusted per 2000 kcal (8368 kJ))						
	T1 (*n* 252)		T2 (*n* 252)		T3 (*n* 251)		
	Mean	sd	Mean	sd	Mean	sd	*P*
Energy density	1·31	0·25	1·22	0·21	1·08	0·18	<0·0001*
NRF_9.3_	553·08	105·47	591·92	88·30	649·80	77·11	<0·0001*
LIM	99·70	43·01	96·82	42·73	86·59	35·09	0·001*
NR9	654·26	70·89	688·74	64·84	736·38	67·69	<0·0001*
HEI-2015	62·57	9·91	67·18	9·70	71·55	7·87	<0·0001*

	Tertiles of total monthly food expenditure						
	T1 (*n* 252)		T2 (*n* 271)		T3 (*n* 232)		
	Mean	sd	Mean	sd	Mean	sd	*P*
Energy density	1·20	0·23	1·22	0·23	1·18	0·25	0·138
NRF_9.3_	588·73	104·25	598·14	90·56	608·54	102·72	0·090
LIM	97·06	41·84	91·51	36·20	94·82	44·48	0·293
NR9	687·28	76·57	689·65	72·20	703·35	77·91	0·042*
HEI-2015	65·23	9·53	66·80	10·04	69·45	9·68	<0·0001*

NRF_9.3_, Nutrient Rich Food Index 9.3; LIM, nutrients to limit; NR9, nine nutrients to encourage; HEI-2015, Healthy Eating Index 2015.

* Statistically significant *P* values.

The results of multiple regression analysis between education, residential property values, two measures of food spending and percentage energy from UP food are shown in Table 4. Models were adjusted for variables in the table, as well as for sex, age and race/ethnicity. In model 1, higher diet cost was also associated with an 11 % less percentage energy from UP foods (*β* = −10·89, 95 % CI −12·66, −9·12). Having college education or higher was associated with 4 % less energy from UP foods (*β* = −4·12, 95 % CI −6·52, −1·73) as compared with high school or less. In adjusted model 2, higher food expenditures and college education were associated with a reduction in percentage energy from UP foods of 3 and 5 %, respectively.

**Table 4.** Linear regression analysis showing association of socio-demographic indicators with percentage energy from ultra-processed foods† (Mean values and 95 % confidence intervals)

	Model 1: Diet cost per 2000 kcal (8368 kJ) ($/month)			Model 2: Total food expenditures per capita ($/month)		
	Mean difference in diet cost	*P*	95 % CI	Mean difference in diet cost	*P*	95 % CI
Education						
High school or less	Ref.			Ref.		
Some college	−1·07	0·331	−3·24, 1·09	−1·75	0·156	−4·18, 0·67
College graduate/graduate school	−4·12	0·001*	−6·52, −1·73	−4·97	<0·0001*	−7·63, −2·31
Residential property value						
Tertile 1 (≤$128 675)	Ref.			Ref.		
Tertile 2 ($128 676–$290 866)	−1·29	0·153	−3·06, 0·48	−1·91	0·071	−3·99, 0·17
Tertile 3 (≥$290 867)	−2·07	0·067	−4·29, 0·15	−3·11	0·017*	−5·67, −0·55
Total monthly food expenditure per capita						
≤$144				Ref.		
≥$145 to ≤250				−1·20	0·168	−2·90, 0·50
≥$251				−3·01	0·007*	−5·20, −0·83
Diet cost per 2000 kcal (8368 kJ) ($/month)						
≤$252·7	Ref.					
≥$252·8 to ≤299·9	−3·78	<0·0001*	−5·32, −2·22			
≥$300	−10·89	<0·0001*	−12·66, −9·12			

* Statistically significant at *P* < 0·01, *P* < 0·0001.

† Both models controlled for all variables in the table as well as sex, age, race/ethnicity and county.

## Discussion

The mean percentage of dietary energy from UP foods in the SOS III sample was 59·7 %, close to the value of 58 % previously calculated from 24-h dietary recalls in the much larger and nationally representative National Health and Nutrition Examination Survey (2007–2012)^(1,32)^. However, there were significant differences by subgroup. Percentage energy from UP foods varied by food expenditures, diet cost and participant SES.

The inverse relation between percentage energy from UP foods and participant education and incomes has been noted before. Past analyses of dietary intakes in National Health and Nutrition Examination Survey 2007–2012 also showed that the consumption of UP foods decreased with age, education and income^(1,32)^. In Canada, UP foods accounted for 47·7 % of energy, with higher percentages reported among groups with lower education (51·7 %)^(33)^. In France, UP foods contributed 35·9 % of total energy intake^(34)^, with higher UP consumption associated with younger age and lower education^(34)^. In the UK^(35)^, data from the National Diet and Nutrition Survey (2008–2012) showed lower consumption of minimally processed foods among lower SES groups, but the consumption of UP foods did not vary by occupational social class, an unexpected finding.

Based on published papers, the direction of the social gradient for UP foods appears to be reversed on low- and middle-income countries. First, socio-economic gradients in diet quality are now apparent in both high-income and in low- and middle-income countries^(36)^. Those associations also hold for children and adolescents^(37)^. When it comes to UP foods, data from Brazil^(38)^ and from Chile^(39)^ have shown that higher percentage energy from UP foods was associated with higher, rather than with lower, SES. In Mexico too^(40)^, higher consumption of UP foods was associated with higher SES and higher education of head of household. Clearly, there are major socio-demographic differences in UP food consumption patterns between the low- and middle-income countries in Latin and in South America and high-income countries such as the USA, Canada, the UK and France.

Given the opposing social gradients in UP foods energy, it is surprising that no study on the relation between UP foods and health has addressed the disparity in food prices and diet costs. In the present study, we used two measures of food spending: food expenditures obtained through self-report and estimated individual-level diet costs. The present method of attaching retail food prices to dietary intakes data from FFQ or from 24-h recalls has now become a standard procedure in nutritional epidemiology^(41)^. In effect, retail costs per 100 g, edible portion, are treated as another nutrient vector. It is important to note that the calculated diet cost reflects the intrinsic monetary cost of the diet and not actual food expenditures. However, both methods produced comparable results.

Efforts to reduce percentage energy from UP foods at the population have made little mention of the economics of food choice. A study from Spain^(42)^ found that an isoenergetic substitution of UP food with unprocessed or minimally processed foods was associated with a significant drop in mortality. At least 28 % of lives would be saved (on paper) if the current consumption of UP foods could be reduced from the highest quartile (68 % of energy) to the lowest quartile (48 % of energy)^(42)^. Whether such a reduction would be associated with a higher per energy diet cost to the affected consumer was not mentioned.

The present analyses can provide some answers. In the present sample, a reduction in the consumption of UP foods from 67·5 % of energy (1st decile) to 48 % of energy (10th decile) was associated with a mean increase in estimated diet costs from $216·4 (1st decile) to $369·9 (10th decile). A $153 increase in monthly diet costs would translate into $7368 per year for a family of four. Clearly, there are economic reasons for why lower-income people select low-cost energy-dense foods as opposed to the recommended ‘prudent’ options. What is not clear is that the association between social class and diet quality^(16,17)^ can be manipulated at will. In the present data, a 10 % drop in energy from UP foods was also associated with an additional $45 169·7 increase in mean residential property values at the tax parcel level.

For the most part, studies have tended to view the consumption of UP foods as a matter of individual choice. Researchers have taken the position that continuing promotion of fresh and minimally processed foods should be the main policy to improve global public health^(13)^. Suggestions for public health interventions generally ran towards consumer education, improved labelling, taxation and marketing restrictions^(13)^. It may be time to focus attention on why people turn to low-cost energy-dense foods in times of economic hardship and stress.

The present confirmation that percentage energy from UP foods is associated with lower food spending and lower energy-adjusted diet costs has some troubling implications for nutritional epidemiology. What observational studies have taught us is that diets associated with lower non-communicable disease risk cost more^(16,43,44)^, whereas diets associated with higher non-communicable disease risk generally cost less^(16–18)^. In other studies, foods associated with weight loss tended to be more expensive^(45)^, whereas foods associated with weight gain were relatively cheap^(45)^. Diets higher in added sugars and saturated fats that have been linked with a higher risk of heart disease^(46)^, obesity and diabetes^(47)^ generally cost less than diets that are unprocessed and nutrient rich.

The present study had limitations. First, the estimates of diet quality indicators were based on FFQ which may lead to bias. However, it is a useful tool to make comparisons across subjects and has been widely used in nutritional epidemiological studies. Second, diet cost estimates do not represent actual expenditures made by the study sample. This limitation has been corrected by asking participants to self-report actual grocery and eating out expenditures. Third, ambiguity in the definition of NOVA classification may have resulted in some misclassification, though this has been minimised by employing two independent researchers to assign food items. Lastly, the present study was based on cross-sectional data; hence, associations observed between SES, diet cost and other diet quality indicator cannot be causally interpreted.

Despite these limitations, the present study has several strengths. This is one of the very few studies to explore the low cost of UP foods in relation to diet quality metrics. The study included the analysis of individual food intake for a cohort of adults from different geographic locations of WA State using a validated FFQ to measure food consumption over a 12-month period.

### Conclusion

Percentage energy from UP foods and measures of food spending were inversely linked. Low-cost foods of high energy density and low nutritional value that are selected by lower-income groups have long been associated with adverse health outcomes. Studies on socio-economic determinants of health would do well to take food prices and affordability into account.
